# Assessment of Nitrite Content in Vienna Chicken Sausages Using Near-Infrared Hyperspectral Imaging

**DOI:** 10.3390/foods12142793

**Published:** 2023-07-23

**Authors:** Achiraya Tantinantrakun, Anthony Keith Thompson, Anupun Terdwongworakul, Sontisuk Teerachaichayut

**Affiliations:** 1 Department of Food Science, School of Food-Industry, King Mongkut’s Institute of Technology Ladkrabang, Chalongkrung Road, Ladkrabang, Bangkok 10520, Thailand; 65086032@kmitl.ac.th; 2 Department of Postharvest Technology, Cranfield University, College Road, Cranfield, Bedford MK430AL, UK; keiththompson28@yahoo.com; 3 Department of Agricultural Engineering, Faculty of Engineering at Kamphaeng Saen, Kasetsart University, Kamphaeng Saen, Nakhon Pathom 73140, Thailand; fengant@ku.ac.th; 4 Department of Food Process Engineering, School of Food-Industry, King Mongkut’s Institute of Technology Ladkrabang, Chalongkrung Road, Ladkrabang, Bangkok 10520, Thailand

**Keywords:** quality control, calibration, prediction, classification, models

## Abstract

Sodium nitrite is a food additive commonly used in sausages, but legally, the unsafe levels of nitrite in sausage should be less than 80 mg/kg, since higher levels can be harmful to consumers. Consumers must rely on processors to conform to these levels. Therefore, the determination of nitrite content in chicken sausages using near infrared hyperspectral imaging (NIR-HSI) was investigated. A total of 140 chicken sausage samples were produced by adding sodium nitrite in various levels. The samples were divided into a calibration set (n = 94) and a prediction set (n = 46). Quantitative analysis, to detect nitrate in the sausages, and qualitative analysis, to classify nitrite levels, were undertaken in order to evaluate whether individual sausages had safe levels or non-safe levels of nitrite. NIR-HSI was preprocessed to obtain the optimum conditions for establishing the models. The results showed that the model from the partial least squares regression (PLSR) gave the most reliable performance, with a coefficient of determination of prediction (R_p_) of 0.92 and a root mean square error of prediction (RMSEP) of 15.603 mg/kg. The results of the classification using the partial least square-discriminant analysis (PLS-DA) showed a satisfied accuracy for prediction of 91.30%. It was therefore concluded that they were sufficiently accurate for screening and that NIR-HSI has the potential to be used for the fast, accurate and reliable assessment of nitrite content in chicken sausages.

## 1. Introduction

Sausages are a food commonly consumed throughout the world, especially as a staple that can be consumed for breakfast, lunch or dinner. There is an increasing demand, throughout the world, for convenient ‘ready-to-eat’ foods [1]. Vienna sausage is one type of popular sausages that can be prepared from many types of meat, including beef, turkey, chicken and pork, by parboiling them and then smoking them at low temperatures, and the production of Vienna sausages is also increasing to supply supermarkets. Additives are commonly used in sausages, mainly to help prevent the growth of harmful bacteria and spoilage microorganisms [2], including sodium nitrite [3]. However, Ludlow et al. [4] reported that the consumption of sausage with excessive preservatives can be detrimental to consumers, and in extreme cases, it can cause diseases and disorders, including methemoglobinemia that can increase the concentration of methemoglobin in the blood. Methemoglobinemia occurs when the hemoglobin is oxidized to methemoglobin because of the ingestion of oxidizing agents. That is, the normal ferrous state (Fe^2+^) of iron in the blood is oxidized to the ferric state (Fe^3+^), resulting in reduced oxygen transportation. Sodium nitrite is a strong oxidizing agent that can cause the formation of methemoglobin [5]. This abnormal condition makes oxygen insufficient in the body’s tissues and organs, which can result in a risk of complications in the body’s tissues and the risk of developing cancers and even death. Pregnant women have been shown to be more susceptible to methemoglobinemia [2]. Safe Food Advocacy Europe [6] reported that consuming processed meat containing nitrites could increase the risk of developing colorectal cancer, and a standard regulation for limiting the addition of nitrites to meat products was limited to a maximum of 80 mg/kg [7]. There are many reports of the toxic effects of consuming sausages containing over the standard level of additives. These include Saengprakay [8], who reported that there were four patients that were hospitalized with methemoglobinemia from nitrite ingestion after consuming sausages without the Thai Food and Drugs Administration approval stamp. Nation News Thailand [9] also reported that 14 children were affected after consuming sausages for the same reason. A disregard for regulating the addition of preservatives in sausage by producers can seriously affect consumers. There are standard analytical methods, such as an ion exchange chromatography and a colorimetric method [10], that have been used for the determination of sodium nitrite in meat products, but those techniques are destructive, more complicated and are time consuming. Therefore, a nondestructive technique to detect nitrite content in sausages, which is fast, reliable and accurate, would be of major benefit to the food industry and to consumers.

Near infrared spectroscopy (NIRS) has been successfully used as a nondestructive technique for measuring various characteristics of food. The vibration energy of hydrogen bonds (X − H), X = C, O, N and S, in the molecule of the sample affected by the NIR light (800–2500 nm) is considered the absorption energy [11]. The near infrared absorption levels of the sample at different wavelengths that appear in the NIR spectra are used for quantitative and qualitative analyses. NIR-HSI is a combination of near infrared spectroscopy and computer vision to acquire the NIR spectral information in each pixel of the image. Therefore, a hyperspectral image is the acquired image in which spectral information is contained in each pixel of the image continuously [12]. Then, the spectral information of a sample from NIR-HSI is obtained in the larger area when compared with NIRS.

NIRS has been used to detect specific elements within the composition in foods, including the salt content in marinade and in fish [13] and the Fe, Ca, K and Na in commercial hamburgers [14]. There are many other studies in which NIR-HSI has been applied to various quality assessments of meat products. These include the detection of offal adulteration in ground pork [15], the prediction of the bacterial growth on beef [16], the prediction of total volatile basic nitrogen (TVB-N) and total viable count (TVC) of bacteria in chicken [17] and the detection of low-cost protein-source adulteration in pure fishmeal (PFM) [18]. Furthermore, several examples of NIR-HSI application have been successfully demonstrated for the prediction of adulteration in food, including fruit and vegetables, such as the detection of limestone powder in tapioca starch [19], the detection of peanut shells, pecan shells and walnut shells in cumin powder [20] and the detection of metanil yellow adulteration in chickpea flour [21]. The advantages of NIR-HSI include that it can detect internal components in every part of a sample using only a single scan, as has been demonstrated in predicting the maturity index of intact pineapples [22] and in the detection of total soluble solids and moisture content in longans [23]. Additionally, NIR-HSI is fast, convenient, reliable and does not require the use of chemicals, thus being environmentally friendly.

Therefore, the aim of this study was to test if NIR-HSI could be successfully utilized to test for nitrite levels in Vienna chicken sausages in order to establish quantitative and qualitative models for predicting nitrite content and classifying the sausages into either safe or not safe. Vienna chicken sausages were selected for this study because of their popularity in Thailand as well as worldwide. Nitrite is normally added to Vienna chicken sausages during processing, which may be harmful to some consumers, especially if residual levels exceed legally permitted levels. Additionally, the products must be specific, such as the Vienna chicken sausages in this study, because the boundary must be controlled in order to accept the results of the prediction accuracy of both the calibration and classification models.

## 2. Materials and Methods

### 2.1. Preparation of Samples

Samples of Vienna sausages were prepared, using the method described by Savic [24], consisting of chicken breast (1000 g), sugar (7.5 g), pepper (2.5 g), sodium erythorbate (5 g), egg white powder (7.5 g), monosodium glutamate (2.5 g) and ice (300 g). The ingredients were thoroughly mixed and then ground, using a cutter mixer ( SIRMAN SpA, Curtarolo, Italy). Sodium nitrite (KEMAUS, Bangkok, Thailand) was then added to the samples in various levels by increasing every 10 mg/kg in each sample. After thoroughly mixing, each sample was filled into a separately prepared lamb intestine with a diameter of 18 mm using an electric meat casing machine (JAO KHUMTHONG, Rayong, Thailand).

### 2.2. Instrumentation

**Figure 1:**
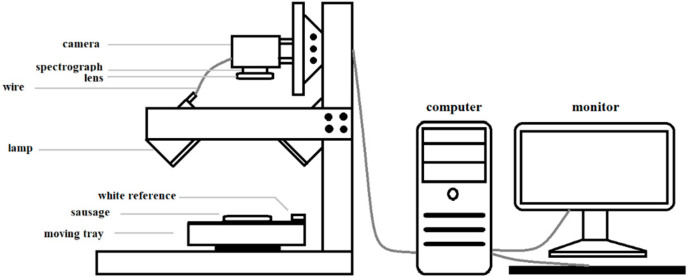
Sample presentation and schematic drawing of the NIR-HSI unit.

A Reflectance Near Infrared Hyperspectral Imaging Unit (Fx17, Spectral Imaging Ltd., Oulu, Finland.) in the wavelength range of 935–1720 nm with a spectral resolution of 3.5 nm was used to acquire spectral images of each sample at a controlled temperature of 25 °C. The total pixel count from the NIR-HSI was 933 × 640 pixel. Each sample of prepared sausage was then placed on the moving tray of the NIR-HSI unit at a speed of 15 mm/s and scanned with an integration time of 30 ms (Figure 1). A white reference image was acquired by scanning a Spectralon directly followed by scans of each sample. The dark reference image was obtained when the light sources were turned off and the lens was covered with a black lid. Each sample was scanned individually to acquire its spectral image.

### 2.3. Residual Nitrite Content Determination

After scanning each sample, using the NIR-HSI, the actual residual nitrite content was determined using the method (AOAC 973.31) [10]. A sodium nitrite standard solution was used in order to create a calibration curve as follows: The nitrite solution was prepared, and then a sulphanilamide reagent was added. After 5 min, a N-(1-Naphthyl) ethylenediamine dihydrochloride (NED) reagent was added. NED is widely used in quantitative analyses of nitrate by colorimetry because of its strongly colored azo compound, and then it was mixed together for about 15 min until the color of the solution developed. The acquired solution was placed in a photometer cell, and its color was determined at a wavelength of 538 nm using a UV-VIS spectrophotometer (Ensight, PerkinElmer Inc., Waltham, MA, USA) against a blank containing water, sulphanilamide reagent and NED reagent. A sodium nitrite solution was prepared from each sausage by adding water, and then it was heated up to 80 °C for 2 h in a shaking steam bath, cooled and filtered to acquire a clear solution. Sulphanilamide and sodium nitrite were then added and left for 5 min, and NED reagent was added and mixed together for about 15 min until the color of the solution developed. The acquired solution was placed in a photometer cell and the color absorption value was determined at a wavelength of 538 nm using the UV-VIS spectrophotometer against the reagent blank, and the residual nitrite content of each sample was determined using the calibration curve from the sodium nitrite standard solution.

### 2.4. Spectral Data of Sausages

**Figure 2:**
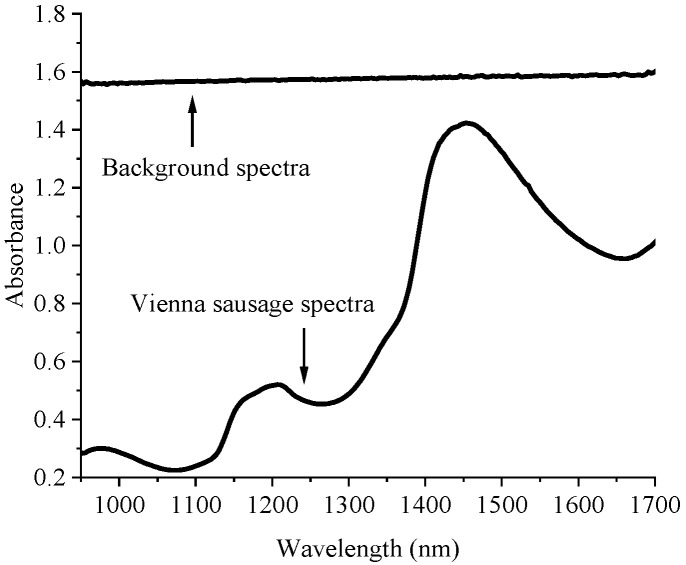
The average spectra of the background and the average spectra of the Vienna sausages from the NIR-HSI scan.

Both the sample image and the background image were acquired from the NIR-HSI scan, which showed that the absorbance spectra of the background and the absorbance spectra of the Vienna sausages were completely different (Figure 2).

Only the spectral image of each sample was selected by removing the spectral image of the background using principal component analysis (PCA). The components of the sample and the background were different; therefore, the spectral information of the sample and the background were different, which means that the spectral image of the background could be separated, as previously described by Bai et al. [25]. Only the remaining spectral image of each sample was used as the region of interest (ROI) for analysis.

### 2.5. Data Analysis

Acquired spectra from the ROI of each sample were averaged and used as its representative and then divided into 2 groups. One group was used for establishing the models, the calibration set (n = 94), and the second group was used for testing the models, the prediction set (n = 46). The spectra of samples were preprocessed in order to select the optimal conditions for developing the models using the following: Savitzky–Golay smoothing, first derivative differentiation (1st derivative), second derivative differentiation (2nd derivative), multiplicative scatter correction (MSC) and standard normal variate transformation (SNV). For quantitative analysis, the calibration model was established using the dataset of spectra as independent variables and nitrite concentration as the dependent variables using partial least squares regression (PLSR) and support vector machine regression (SVMR). The accuracy of the model was determined using the coefficients of correlation of prediction (R_p_) and the root mean square error of prediction (RMSEP), as previously described by Emil W. Ciurczak [11]. For qualitative analysis, principal component analysis (PCA) was used to evaluate the differences between groups of samples that contained unsafe nitrite concentrations compared to safe nitrite concentrations. The classification analysis was performed using partial least square-discriminant analysis (PLS-DA) and support vector machine classification (SVMC). The classification of sausages based on nitrite concentration levels at the cutoff value of 80 mg/kg was determined. Samples that contained nitrite concentrations equal to or over 80 mg/kg were given a value of 0, while samples that contained nitrite concentrations under 80 mg/kg were given a value of 1. The accuracy of the classification models was investigated using a percentage of samples where the prediction was correct.

The data were analyzed by Microsoft Office Excel 2016, the Umbio Evince Hyperspectral Imaging Software (Prediktera Evince, version 2.7.5, Umea, Sweden) and the Unscrambler X version 10.5.1 (CAMO, Oslo, Norway) in this study.

## 3. Results and Discussion

### 3.1. Calibration Curve of the Nitrite Standard Solution

**Figure 3:**
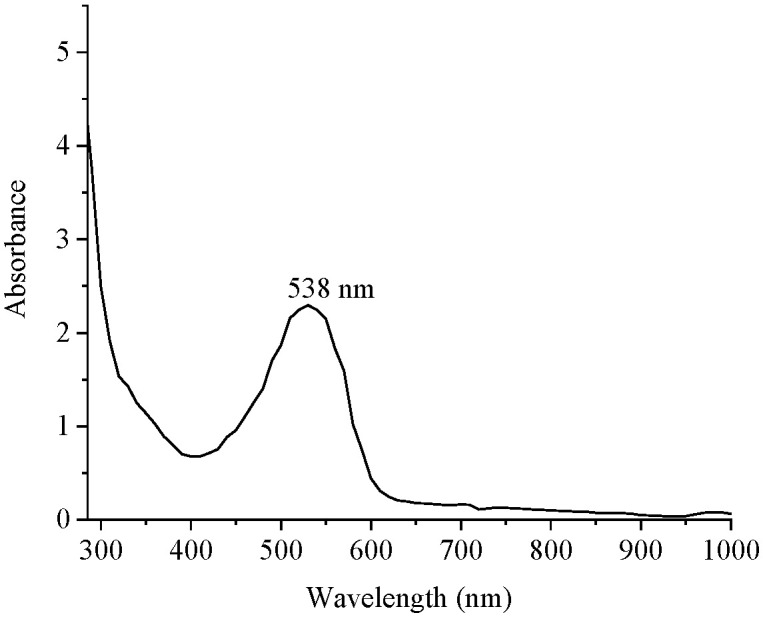
Spectrum of nitrite standard solution from a UV-VIS spectrophotometer.

The calibration curve of nitrite concentration by nitrite standard solution using a UV-VIS spectrophotometer was created based on the official AOAC 973.31 method. The feature of the spectrum of the nitrite standard solution contained an absorption peak at 538 nm (Figure 3). The equation for determining nitrite content at the absorption peak of 538 nm was y = 1.817x + 0.1496, and the coefficient of determination (R^2^) of the equation was 0.993.

### 3.2. Spectral Data of Sausage

**Figure 4:**
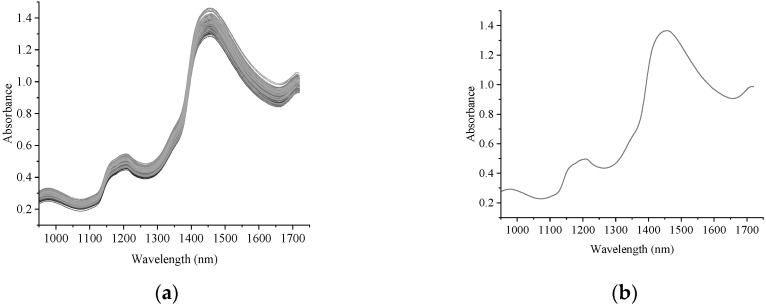
The absorbance spectrum of all chicken Vienna sausages (**a**) and the average absorbance spectrum of chicken Vienna sausages (**b**).

There were absorbance peaks at around 1208 and 1460 nm of the absorbance spectra of all chicken Vienna sausages (Figure 4a,b), since the Vienna sausages contained sugar, pepper, sodium erythorbate and monosodium glutamate. The spectra showed an absorption peak at 1208 nm, which has previously been shown to be related to the first overtone of C–O stretching and second overtone of C–H stretching [26]. The Vienna sausages contained chicken breast and egg white, which showed in the spectra of the absorption peaks at 1208 nm and 1460 nm. These peaks were previously shown to be related to the second overtone of N–H stretching and first overtone of N–H stretching, respectively [27].

### 3.3. Quantitative Analysis

The characteristics of the residual nitrite content of samples showed that the distribution of data in both the calibration set and the prediction set were similar (Table 1), and the residual nitrite content of samples in the prediction set was covered with those of data in the calibration set.

The spectral data of samples in the calibration set were preprocessed in order to determine the optimal conditions for creating the calibration models using PLSR and SVMR (Table 2). SNV spectral pretreatment gave the best results compared with others. The results showed that the PLSR model obtained a higher coefficient of correlation of cross-validation (R_cv_) and lower root mean square error of cross-validation (RMSECV) when compared with the SVMR model (R_cv_ = 0.897 and RMSECV = 17.57 mg/kg). Therefore, SNV spectral pretreatment and PLSR were selected for developing the calibration model in this study. The accuracy of the PLSR model was evaluated by testing the calibration model with samples in the prediction set (N = 46). The results showed that the prediction accuracy was satisfactory with an R_p_ of 0.920 and RMSEP of 15.60 mg/kg (Table 3).

**Figure 5:**
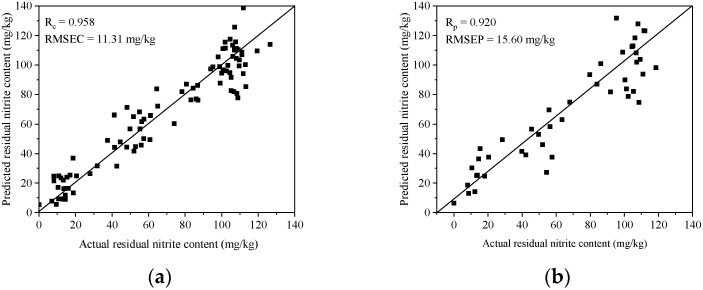
The scatter plot of predicted values and actual values of residual nitrite content in samples using the partial least squares regression model in both the calibration set (**a**) and the prediction set (**b**).

The results of the predicted values and actual values of the residual nitrite content in samples in the calibration set and the prediction set (Figure 5a and Figure 5b, respectively) show that the cluster of scatter plots closely approached the 45° line. This implies that the model showed satisfied accuracy for predicting residual nitrite content in the samples.

### 3.4. The Visualization of Nitrite Content in Sausage

**Figure 6:**
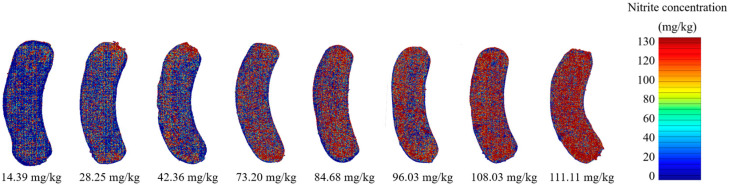
Predictive images of Vienna chicken sausage based on residual nitrite concentration.

In application, the acquired calibration model could be used for creating predictive images that were used for the visual inspection of sausages based on the level of nitrite content. Each pixel of the spectral image of the scanned sample from NIR-HSI was used to interpret the nitrite content by the calibration model to a color-distribution-based image. In this step, the PLSR model with SNV spectral pretreatment was used to predict the nitrite concentration in each pixel of each spectral image of the sausage that had been acquired from NIR-HSI. The visualized image was created for presenting the level of nitrite content in every area of each sausage that correlated between nitrite concentration and different color (Figure 6). The predictive images showed clear different colors from blue (the lowest amount of residual nitrite content) to red (the highest amount of residual nitrite content) based on the color scale related to the residual nitrite concentration in the sausage. These images also showed the distribution of nitrite concentration in each sample.

### 3.5. Qualitative Analysis

**Figure 7:**
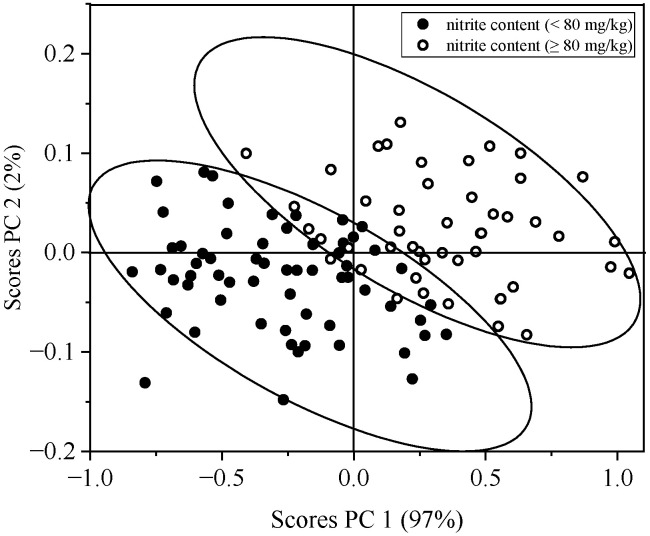
PCA of spectral data of safe nitrite level samples (<80 mg/kg) and unsafe nitrite level samples (≥80 mg/kg).

The plot of the scores for the first principal component (PC1) and the second principal component (PC2) based on the spectral data of unsafe nitrite level samples and safe nitrite level samples showed two clusters with variations for PC1 and PC2 of 97% and 2%, respectively. The cumulative variance percentage was 99% for PC1 and PC2. The result of the score plot of PC1 and PC2 showed that the two clusters were not completely separated, and there was an overlap between unsafe nitrite level samples and safe nitrite level samples (Figure 7).

For establishing the classification model, samples were separated into two groups (0, 1). The first group was the samples (n = 70) that contained safe levels of residual nitrite content (<80 mg/kg), while the second group spectra included samples (n = 70) that contained unsafe levels of residual nitrite content (≥80 mg/kg), and then, samples from two groups were divided into the calibration set and prediction set. The distributions of nitrite content in both the calibration set and the prediction set were similar (Table 4).

Average spectral data from the ROI of samples in both groups in the calibration set were preprocessed in order to investigate the optimal conditions for classification analysis using PLS-DA and SVMC (Table 5), which showed that MSC and SNV spectral pretreatments gave the best results for PLS-DA and SVMC, respectively. The best total accuracy of classification was 98.94% by PLS-DA. Therefore, the MSC spectral pretreatment was selected for developing the classification model using PLS-DA. The accuracy of the classification model was evaluated by testing the classification model with samples in the prediction set. The results showed that the classification accuracy in the prediction set by the classification model was 91.30% (Table 6).

**Figure 8:**
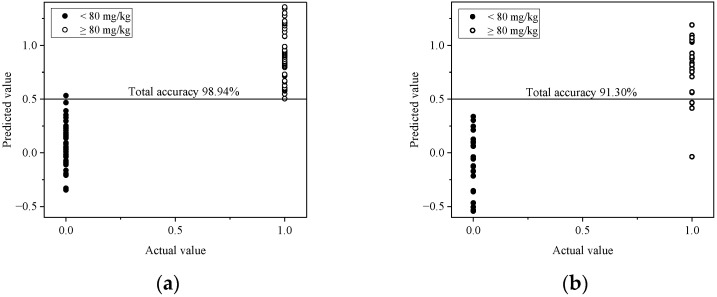
The scatter plot of predicted values and actual values for the group classification of 0 (<80 mg/kg) and 1 (≥80 mg/kg) in the calibration set (**a**) and the prediction set (**b**).

The results of classification by PLS-DA, shown by the scatter plot of predicted and actual values of the samples (0), contained safe levels of residual nitrite content (<80 mg/kg), and the samples (1) contained unsafe levels of residual nitrite content (≥80 mg/kg) in the calibration set (Figure 8a) and in the prediction set (Figure 8b) with a cut off value of 0.5.

Results in this study correspond to the recent reports that the NIR-HSI technique is non-destructive, fast, reliable and accurate to evaluate the qualities of products [28,29,30,31]. The calibration model and the classification model, which were established using the NIR-HSI technique, showed good accuracy for predicting the nitrite content in Vienna sausages and classifying Vienna sausages for safe nitrite levels (<80 mg/kg) and non-safe nitrite levels (≥80 mg/kg). These results indicate that NIR-HSI could be used for both quantitative and qualitative analyses, which correspond to the report of Sricharoonratana et al. [32], who found that NIR-HSI could be successfully used for predicting the storage time of cakes and classifying expired and non-expired cakes based on microorganism infections during storage. Additionally, the report of Sahachairungrueng et al. [33] showed that NIR-HSI could be successfully used for determining the concentration of Robusta in a mixture of Arabica and Robusta coffee and classifying the respective levels of Arabica and Robusta in a roasted ground coffee mixture. In other works, Badaró et al. [34] showed that NIR-HSI could be successfully used for determining the pectin content of orange peel and classifying it into groups based on pectin content; Kucha et al. [35] showed that NIR-HSI could be used to evaluate the iodine content of pork fat and classifying pork subcutaneous fat from different locations of the pig carcass; Talens et al. [36] showed that NIR-HSI could be used to predict the water and protein contents of Spanish cooked ham and classify the hams into different quality categories.

## 4. Conclusions

Using NIR-HSI to acquire the average spectra, from a region of interest within the wavelength of 935–1720 nm of sausages made from chicken was used to determine their nitrite content. The PLSR model with SNV spectral pretreatment was established for quantitatively predicting the level of nitrite in the sausages, which had a coefficient of determination of prediction of 0.92 and a root mean square error of prediction of 15.60 mg/kg. The acquired model was successfully used to create predictive images that could be used to visually determine their nitrite concentration based on a color scale. The classification results from PLS-DA with the MSC spectral pretreatment method gave a total prediction accuracy of 91.30%. This shows that NIR-HSI could be used for the successful classification of individual sausages in terms of it conforming, or not conforming, to the safe nitrite level as laid down by the FAO and WHO Food Standard [3]. Another advantage of NIR-HSI is that it is fast and non-destructive compared to the official AOAC 973.31 method, and as such, it can be applied for use in online grading and inspection systems in factories, where residual nitrite levels of each sausage on a conveyer can be inspected in real time. However, there are limitations in the application of NIR-HSI to online sorting and grading systems, mainly because the unit is quite expensive. The payback period to recover the cost of the investment would be based on the quantity of production and could have the advantage of reduction labor, chemical use and industrial waste, as well as speeding up operations. Therefore, it was concluded that NIR-HSI has the potential to be used in assessing and classifying the level of nitrite in Vienna chicken sausages that could be applied to online systems in the factory, but further work is needed, especially in terms of its economic viability.

## Figures and Tables

**Table 1:** The dependent variable of Vienna chicken sausage samples for establishing the calibration model.

Variable	Descriptions	Calibration	Prediction
Residual nitrite content	Number of samples	94	46
	Range (mg/kg)	0–126.4	0–118.5
	Average (mg/kg)	65.42	65.46
	Standard derivation (mg/kg)	39.87	39.49

**Table 2:** Spectral pretreatment methods for establishing the calibration model.

No.	Pre-Processing Techniques	PLSR				SVMR		
		N	F	R_cv_	RMSECV (mg/kg)	N	R_cv_	RMSECV (mg/kg)
1	Original	94	11	0.897	17.62	94	0.599	32.05
2	Smoothing	94	11	0.889	18.24	94	0.589	32.41
3	1st Derivative	94	6	0.869	19.77	94	0.791	24.49
4	2nd Derivative	94	7	0.799	24.23	94	0.659	0.71
5	MSC	94	9	0.882	18.78	94	0.800	23.98
6	SNV	94	9	0.897	17.57	94	0.800	23.97
7	Smoothing + 1st Derivative	94	8	0.890	18.12	94	0.765	25.67

F = factor, R_cv_ = coefficients of correlation of cross-validation, RMSECV = root mean square error of cross-validation.

**Table 3:** PLSR model for predicting nitrite content in sausages.

Analytical Method	Pre-Treatment	Calibration			Prediction		
		N	R_c_	RMSEC (mg/kg)	N	R_p_	RMSEP (mg/kg)
PLSR	SNV	94	0.958	11.31	46	0.920	15.60

R_c_ = coefficients of correlation of calibration, RMSEC = root mean square error of calibration, R_p_ = coefficients of correlation of prediction, RMSEC = root mean square error of prediction.

**Table 4:** Residual nitrite content in Vienna chicken sausages in the calibration set and prediction set.

Parameter	Items	Calibration		Prediction	
Nitrite content		Group 0 (<80 mg/kg)	Group 1 (≥80 mg/kg)	Group 0 (<80 mg/kg)	Group 1 (≥80 mg/kg)
	Number of samples	47	47	23	23
	Range (mg/kg)	0–78.14	81.00–126.40	0–79.64	83.89–118.49
	Average (mg/kg)	32.02	80.60	33.53	103.47
	Standard derivation (mg/kg)	22.49	9.55	23.16	8.53

**Table 5:** Results of the pre-processing techniques for PLS-DA and SVMC in the calibration set.

Methods			Original	Smoothing	1st Derivative	2nd Derivative	MSC	SNV
PLS-DA	0 (<80 mg/kg)	correct	45/47	46/47	46/47	38/47	46/47	46/47
		incorrect	2/47	1/47	1/47	9/47	1/47	1/47
	1 (≥80 mg/kg)	correct	45/47	46/47	45/47	35/47	47/47	45/47
		incorrect	2/47	1/47	2/47	12/47	0/47	2/47
	Total accuracy		90/94	92/94	91/94	73/94	93/94	91/94
	Total accuracy (%)		95.74	97.87	96.81	77.66	98.94	96.81
SVMC	0 (<80 mg/kg)	correct	38/47	38/47	39/47	14/47	45/47	47/47
		incorrect	9/47	9/47	8/47	33/47	2/47	0/47
	1 (≥80 mg/kg)	correct	25/47	40/47	30/47	43/47	31/47	35/47
		incorrect	22/47	7/47	17/47	4/47	16/47	12/47
	Total accuracy		63/94	78/94	69/94	57/94	76/94	82/94
	Total accuracy (%)		67.02	82.98	73.40	60.64	80.85	87.23

**Table 6:** PLS-DA for classifying safe nitrite level samples (<80 mg/kg) and unsafe nitrite level samples (≥80 mg/kg).

Analytical Method	Pre-Treatment	Calibration		Prediction	
		N	Total Accuracy (%)	N	Total Accuracy (%)
PLS-DA	MSC	94	98.94	46	91.30

## Data Availability

The data presented in this study are available on request from the corresponding author. The data are not publicly available due to large data in many sections in this study.
